# Contactless magnetically responsive injectable hydrogel for aligned tissue regeneration

**DOI:** 10.1016/j.mtbio.2024.101110

**Published:** 2024-06-03

**Authors:** Arianna Rossi, Franco Furlani, Giada Bassi, Carla Cunha, Alice Lunghi, Filippo Molinari, Francisco J. Teran, Florigio Lista, Michele Bianchi, Anna Piperno, Monica Montesi, Silvia Panseri

**Affiliations:** a Institute of Science, Technology and Sustainability for Ceramics, National Research Council of Italy. Via Granarolo 64, 48018. Faenza, Italy; b University of Messina, Department of Chemical, Biological, Pharmaceutical and Environmental Sciences. Viale Ferdinando Stagno d'Alcontres, 31, 98166, Messina, Italy; c University of G. D'Annunzio, Department of Neurosciences, Imaging and Clinical Sciences. Via Luigi Polacchi, 11, 66100 Chieti, Italy; d i3S - Instituto de Investigação e Inovação em Saúde. Rua Alfredo Allen 208, 4200-135, Porto, Portugal; e Center for Translational Neurophysiology of Speech and Communication, Istituto Italiano di Tecnologia 44121 Ferrara, Italy; f Section of Physiology, Università di Ferrara 44121 Ferrara, Italy; g Defense Institute for Biomedical Sciences, IGESAN, Via di Santo Stefano Rotondo 4, 00184 Rome, Italy; h iMdea Nanociencia, Ciudad Universitaria de Cantoblanco, 28049 Madrid, Spain; i Nanotech Solutions, Ctra Madrid23, 40150 Villacastín, Spain; j Department of Life Sciences, Università degli Studi di Modena e Reggio Emilia 44125 Modena, Italy

**Keywords:** Responsive hydrogel, Regenerative medicine, Minimally invasive surgery, Anisotropic architecture, Magnetic biomaterial

## Abstract

Cellular alignment plays a pivotal role in several human tissues, including skeletal muscle, spinal cord and tendon. Various techniques have been developed to control cellular alignment using 3D biomaterials. However, the majority of 3D-aligned scaffolds require invasive surgery for implantation. In contrast, injectable hydrogels provide a non-invasive delivery method, gaining considerable attention for the treatment of diverse conditions, including osteochondral lesions, volumetric muscle loss, and traumatic brain injury.

We engineered a biomimetic hydrogel with magnetic responsiveness by combining gellan gum, hyaluronic acid, collagen, and magnetic nanoparticles (MNPs). Collagen type I was paired with MNPs to form magnetic collagen bundles (MCollB), allowing the orientation control of these bundles within the hydrogel matrix through the application of a remote low-intensity magnetic field. This resulted in the creation of an anisotropic architecture. The hydrogel mechanical properties were comparable to those of human soft tissues, such as skeletal muscle, and proof of the aligned hydrogel concept was demonstrated.

*In vitro* findings confirmed the absence of toxicity and pro-inflammatory effects. Notably, an increased fibroblast cell proliferation and pro-regenerative activation of macrophages were observed. The *in-vivo* study further validated the hydrogel biocompatibility and demonstrated the feasibility of injection with rapid *in situ* gelation. Consequently, this magnetically controlled injectable hydrogel exhibits significant promise as a minimally invasive, rapid gelling and effective treatment for regenerating various aligned human tissues.

## Graphical abstract

**Figure:**
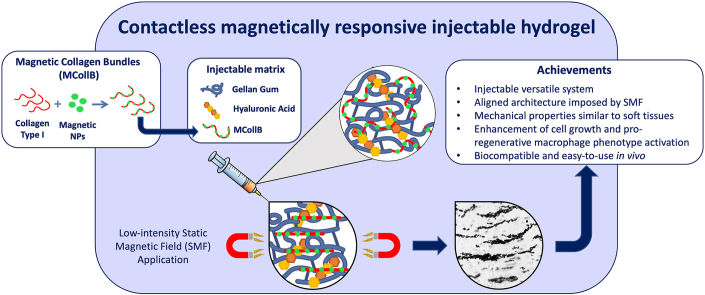

## 1 Introduction

Human tissues have well-defined architectures and mechanical properties that play a crucial role in their functionality. In each tissue/organ, the extracellular matrix (ECM) is an active player since it creates a complex network of proteins and glycosaminoglycans that constantly interact with cells, enhancing their activity and organization [[1], [2], [3], [4], [5], [6]]. The intricate landscape of the cellular microenvironment plays a pivotal role in dictating the course of tissue growth and development. Its nuanced characteristics, encompassing factors like ECM composition, cell-cell interactions, and biochemical signalling, wield a profound influence on the fate and behaviour of cells within a tissue [[7], [8], [9]].

In several traumas and diseases, the cellular component and the cell microenvironment are compromised. The human body has the capability to regrow lost tissues in response to moderate injuries. However, in severe diseases where the loss of cells and ECM is significant (e.g., osteochondral lesion, volumetric muscle loss, traumatic brain injury) [[10], [11], [12]], biomaterial-based approaches could sustain and enhance the regenerative process [13]. Considering the importance of the cell microenvironment in the functionality of tissue-engineered organ substitutes, a customized 3D biomaterial that mimics the physical, chemical, and mechanical architecture of the native ECM is fundamental in supporting cell growth. Specifically, in several tissues and organs such as the spinal cord, muscle, tendon, and cartilage, cellular alignment is fundamental. In these scenarios, the ideal biomaterials should also be able to replicate the anisotropic microarchitecture with well-oriented 3D structures to support regeneration in these specific human anatomical districts. Several scaffolds capable of reproducing crucial tissue alignment and sustaining the regenerative process have been produced using various techniques, including bioprinting, electrospinning, and freeze-drying. Despite promising results, these techniques still have some limitations. For example, 3D bioprinting requires specific features regarding the material selection for the bio-ink, making the produced scaffold in the short term suitable for only certain organs [14]. Furthermore, in the majority of extrusion-based techniques, the light utilized for photopolymerization, which is crucial to harden the bio-ink, often leads to diminished cell viability and displays limited penetration capability. This limitation ultimately results in the stabilization of only the outer layers of 3D-printed materials [[15], [16], [17], [18], [19], [20]]. Conventional electrospinning methods, mainly suitable for synthetic polymers, produce scaffolds characterized by restricted thickness and dense packing, which may result in inadequate cell infiltration [[21], [22], [23], [24], [25]]. The freeze-drying technique is a time-consuming process with high energy consumption, and the fabrication procedure for devising implantable materials with the desired shape is challenging to fine-tune at the nanoscale level [25,26]. In addition, a significant drawback of all these techniques is that the resulting scaffolds are preformed in the laboratory, and quite invasive surgery is needed to transplant them into the target tissue/organ, such as the brain, spinal cord, and muscles. These limitations hinder, their practical application in routine medical treatments [27,28].

On the other hand, hydrogels stand as a promising asset in tissue engineering due to their potential for minimally invasive application, allowing for simple syringe injection into irregularly shaped injury defects. Hydrogels undergo a sol-gel transition in response to various stimuli like temperature and pH, facilitating their adaptability to diverse biological environments. However, an inherent limitation of hydrogels is their inability to spontaneously create well-aligned structures *in situ*, a characteristic essential for mimicking natural tissue architecture [[29], [30], [31], [32], [33], [34]].

To address this limitation, recent studies have delved into the integration of magnetic nanoparticles (MNPs) with polymers to confer anisotropic properties to 3D hydrogels [[32], [33], [34], [35], [36], [37], [38], [39], [40], [41], [42]]. The incorporation of MNPs can occur through straightforward methods like mixing or more sophisticated techniques such as magnetic electrospinning or 3D printing of fibers [35,37,43,44].

This approach aims to introduce controlled alignment within the hydrogel matrix, mimicking the natural orientation of tissues, thereby enhancing their functionality in tissue regeneration and repair processes.

MNPs have displayed distinctive magnetic properties that have been widely used in various biomedical applications, such as magnetic hyperthermia, bioseparation, cell manipulation and cell/drug delivery, contrast agents for magnetic resonance imaging (MRI), and more in general for the development of tissue constructs [[45], [46], [47]].

Taking advantage of MNPs' magnetic features, we combined them with collagen type I fibers (i.e., the most abundant collagen in the human body) to obtain magnetic collagen bundles (MCollB) that could be aligned in response to a low-intensity static magnetic field. MCollB were immersed in gellan gum (GG) matrix used for its easy gelation in the presence of ions that typically exist in any human tissue/organ microenvironment (e.g., Ca^2+^; Mg^2+^; Na^+^; K^+^). Specifically, GG is a water-soluble anionic polysaccharide produced by the bacterium Sphingomonas elodea, which can be easily processed into transparent gels that are resistant to heat and acid stress without the use of harsh reagents [48]. GG is extensively employed in the food industry for its role as a thickening agent or stabilizer. However, due to its well-established biocompatibility, it is currently being investigated for various medical applications including the development of drug delivery systems and tissue engineering approaches [[49], [50], [51]]. To achieve a more biomimetic hydrogel, hyaluronic acid (HA), a glycosaminoglycan abundantly present in human tissues, was combined with GG and MCollB to augment cellular activities such as adhesion, proliferation, and migration.

The resulting hydrogel (GG_HA_MCollB) could be easily injected through a fine needle and was contactless magnetically responsive, allowing the achievement of an anisotropic architecture. This promising matrix was extensively studied, considering rheological, mechanical, magnetic, and biological aspects (cytotoxicity and immune response) both *in vitro* and *in vivo*.

## 2 Material and methods

### 2.1 Materials

Gellan gum (GG, phytagel, Sigma Aldrich), hyaluronic acid bt (HA, molecular weight: 1700 kDa, DSM), trisodium citrate (SC, Merck), Iron (III) oxide nanopowder <50 nm (MNPs, Sigma Aldrich), collagen type I from rat tail (Sigma Aldrich), DC Protein Assay Kit (Bio-Rad), paraformaldehyde (PFA, Sigma Aldrich), phosphate-buffered saline (1X) w/o Ca and Mg (PBS, Gibco), DMEM high glucose (Gibco), calf bovine serum (CBS, ATCC-30-2030), foetal bovine serum (FBS, Gibco), horse serum (Sigma Aldrich), penicillin/streptomycin mixture (pen/strep, Gibco), trypsin 0.5 % EDTA (Gibco), trypan blue (Sigma Aldrich), LIVE/DEAD® (Invitrogen), Presto blue™ (Invitrogen), triton X-100 (Sigma Aldrich), ActinRed 555 ReadyProbes reagent (Invitrogen), 4’,6-diamidino-2-phenylindole dihydrochloride (DAPI, Invitrogen) reagent, lipopolysaccharide (LPS, Sigma Aldrich), tri reagent (Invitrogen), Directzol RNA MiniPrep kit (Zymo Research), TaqMan Gene Expression Assays (Applied Biosystems), xylene (Sigma Aldrich), ethanol (Sigma Aldrich), Mayer haematoxylin (Fluka), acetic acid (Sigma Aldrich), Potassium hexacyanoferrate (II) (Sigma Aldrich), Safranin-O (Sigma Aldrich), Fast Green (Sigma Aldrich), Eosin Y (Sigma Aldrich), mount (Histo-Line), OCT (Histo-Line); were purchased and used without any further purification.

### 2.2 Magnetic set up

**Fig. 1:**
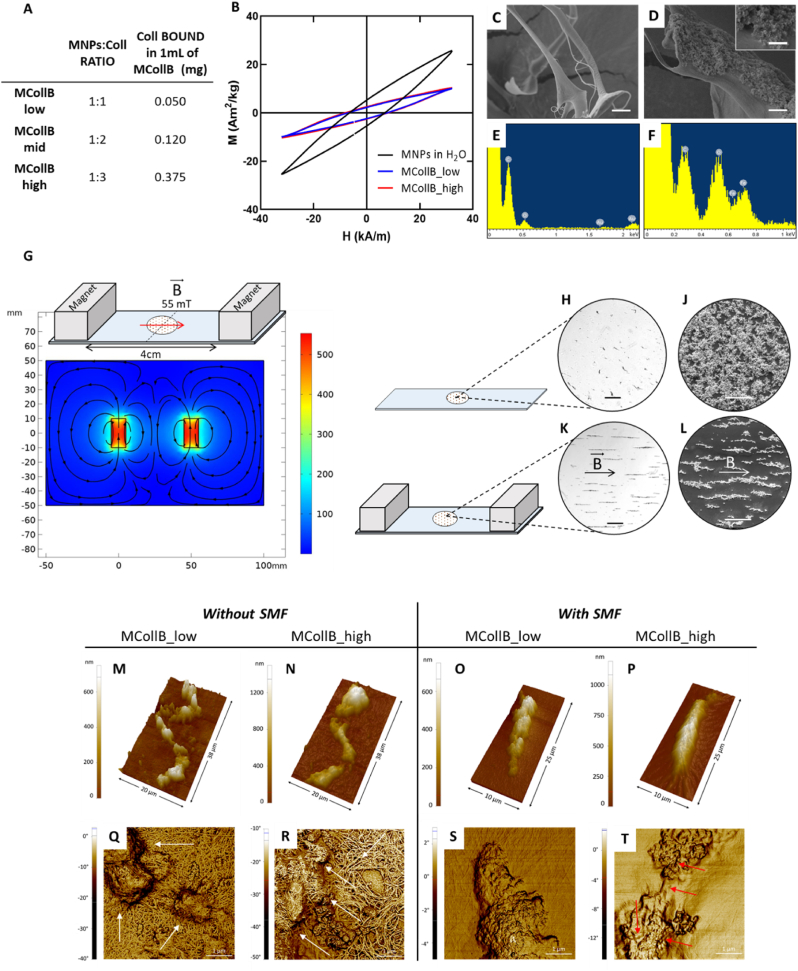
Magnetic collagen bundles (MCollB) characterizations. (A) Collagen content in MCollB. (B) AC magnetization cycles obtained at 20 kHz and 32 kA/m for MNPs, MCollB_low and MCollB_high dispersed in water at 1 g of Fe per liter. (C–F) FEG-SEM images and EDS analyses of the MNPs and collagen interaction: (C) free collagen fibers, (D) MCollB representative image and enlargement (E–F) EDS of free collagen fibers and MCollB respectively. Scale bars: C, D 2 μm; D enlargement 500 nm. (G) Schematic representation of the magnetic system and magnetic flux lines generated by two Neodymium magnets 4 cm apart simulated with COMSOL software. (H–L) Microscope analyses of MCollB_high with or without applying SMF. (H) and (J) optical microscope; (K) and (L) SEM images. In detail, MCollB without SMF in (H) and (J); aligned MCollB after the application of SMF in (K) and (L). Scale bars: H, K 200 μm; J, L 100 μm. (M–T) AFM images showing representative 3D topography (M − P) and phase images (Q–T) of single MCollB. White arrows in (Q) and (R) indicate MNPs aggregates. Red arrows in (T) point out exposed collagen fibers.

Two Neodymium magnets (20 × 10 × 10 mm; magnetic flux density on load point = 767 mT, surface flux density = 460 mT, MagFine srl) were placed at 4 cm distance north pole and south pole facing each other and the samples were always placed in the middle of system (2 cm from each magnet) (Fig. 1 G). The magnetic field measured by a magnetometer at this point was equal to 55 mT. A schematic representation of the magnetic flux generated by the two magnets was generated by COMSOL Multiphysics Software (version 5.4).

### 2.3 Hydrogels preparation

All the hydrogel components were prepared separately. GG was solubilized at a concentration equal to 1.25 % w/v at 70 °C under magnetic stirring using deionized water containing 0.125 % w/v SC as a solvent, obtaining a GG hydrosol. HA was solubilized at 3 % w/v in deionized water under magnetic stirring. MNPs powder was resuspended at a concentration equal to 2.5 % w/v in 95 mM sodium citrate solution and sonicated for 5 min. Magnetic collagen bundles (MCollB) were prepared by mixing MNPs (0.1 % w/v) and collagen type I (0.1 % – 0.3 % w/v) in different weight ratios equal to 1:1 and 1:3, then the pH was adjusted to 7.4. MCollB were then isolated from the supernatant by using a Neodymium magnet (20 × 10 × 10 mm; magnetic flux density on load point = 767 mT, surface flux density = 460 mT, MagFine srl) and resuspended in MilliQ water. A colorimetric assay (DC Protein Assay Kit) was used to determine collagen concentration following the manufacturer's instructions. MCollB were prepared as described above and precipitated using a magnet, the supernatant was removed and quantified. According to this approach, it was possible to determine the amount of collagen bound to MNPs by subtraction (n = 3).

GG, HA, MCollB or free collagen solutions were then combined to obtain hydrosols with different compositions according to Table 1. The gelation of the resulting mixture was then promoted by using cations-containing solutions (i.e. PBS 1X, cell culture media, biological fluids) according to three different approaches: i) injection through a 30G needle into cations-containing solutions ii) casting in 96 well-plate for biological characterizations and iii) casting into discs for stability, rheological and mechanical analyses. In detail the latter approach was done by pouring the hydrosol into a 6 or 12 well-plate coated with 5 layers of paper filter discs previously soaked with PBS 1X, then PBS 1X was sprayed atop and the resulting system was left at rest for 10 min at room temperature (RT), then transferred into a petri dish, submerged in PBS 1X and incubated overnight before rheological and mechanical characterizations.

**Table 1:** Hydrogel formulations (% w/v). Gellan gum (GG), hyaluronic acid (HA), collagen type I (Coll), iron oxide nanoparticles (MNPs), and magnetic collagen bundles (MCollB).

	Gellan Gum	Hyaluronic Acid	Collagen	MCollB	
				MNPs	Coll
GG	1 %				
GG_HA	1 %	0.3 %			
GG_Coll_low	1 %		0.005 %		
GG_HA_Coll_low	1 %	0.3 %	0.005 %		
GG_MCollB_low	1 %			0.1 %	0.1 %
GG_HA_MCollB_low	1 %	0.3 %		0.1 %	0.1 %
GG_Coll_high	1 %		0.0375 %		
GG_HA_Coll_high	1 %	0.3 %	0.0375 %		
GG_MCollB_high	1 %			0.1 %	0.3 %
GG_HA_MCollB_high	1 %	0.3 %		0.1 %	0.3 %

### 2.4 Characterization of the magnetic collagen bundles

#### 2.4.1 Dynamic light scattering analysis

The surface charge (ζ-potential) of free collagen and MNPs - 0.04 mg/mL at pH 7.4 was investigated by means of dynamic light scattering (DLS) using a Zetasizer Nano ZS with 173° detection optics (Malvern Instruments).

#### 2.4.2 Microscopy evaluation

MCollB groups (MCollB_low and MCollB_high) were observed by optical microscopy (Inverted Ti-E Microscope, Nikon) before and after exposing them to a static magnetic field (55 mT). MCollB_low and MCollB_high were also analysed by using Stereoscan 360 SEM (Cambridge Instruments). In detail, they were diluted 1:1 in MilliQ water and a 50 μL drop was poured on a 13 mm diameter glass coverslip and let air dry either with or without a static magnetic field application. The coverslips were placed on aluminium stubs by using an adhesive carbon tape, gold-sputtered by a Polaron Sputter Coater E5100 (Polaron Equipment). A more informative analysis of the interaction between MNPs and collagen fibers was performed by field emission-gun scanning electron microscopy (FEG-SEM) and energy X-ray spectroscopy X-ray spectroscopy (EDS). Briefly, free collagen type I and MCollB_high were freeze-dried (– 40 °C and +25 °C) for 48 h under 0.086 mbar vacuum conditions (5 Pa, LIO 3000 PLT) and then placed on stubs and gold-sputtered as described above. The images and the EDS measurements were acquired by a ΣIGMA FEG-SEM microscope (ZEISS NTS Gmbh).

Details of the arrangement of magnetic collagen bundles (MCollB_low and MCollB_high) with or without the application of the static magnetic field were obtained by Atomic Force Microscopy (AFM) analysis. Different scan size images from 20 × 40 μm^2^ down to 2 × 2 μm^2^ were acquired with a Park XE7 AFM system (Park System) operated in tapping mode in air at room temperature using a pre-mounted silicon cantilever (OMCL-AC160TS, Olympus Micro Cantilever) with Al backside reflective coating. The typical tip curvature radius of the tip ca. 7 nm, elastic constant ca. 26 Nm^−1^ and resonance frequency ca. 300 Hz. Topography and phase images were analysed using Park System XEI software (Park System, Suwon, Republic of Korea) and Gwyddion 2.56 free analysis software.

#### 2.4.3 Magnetic characterization

Magnetization measurements were performed at room temperature testing the MNPs in solution (0.1 % w/v) and added to the collagen to obtain the MCollB (low, high; formulated according to hydrogel preparation section). We employed a commercial inductive magnetometer (SENS AC Hyster™ Series, Nanotech Solutions). AC Hyster Series measures magnetization cycles from MNPs dispersed in different solutions at room temperature under alternating magnetic fields whose frequency ranges from 10 up to 100 kHz and intensities up to 32 kA/m. Each AC magnetization measurement consists of three repetitions to obtain an average of the magnetization cycles and the related magnetic parameters (H_C_, M_R_, AC magnetic hysteresis area). Magnetization units were normalized by the magnetic element mass (i.e. iron or iron plus cobalt magnetic elements) and expressed in Am^2^/kg.

### 2.5 Hydrogel characterization

#### 2.5.1 Stability evaluation

The stability of the formulated systems was carried out on hydrogels obtained according to the hydrogel preparation section (22 mm diameter; 3 mm thickness) incubated in PBS 1X at 37 °C up to 80 days. The weight was measured at different time points: 0 h, 1 h, 3 h, 5 h, 1 d, 3 d, 7 d, 10 d, 14 d, 50 d and 80 d. The data are reported as a percentage respect to the 0 h time point (n = 4).

#### 2.5.2 Rheological characterization

Rheological measurements were carried out on hydrogel discs (prepared according to section hydrogel preparation, 35 mm diameter and 3 mm thickness) using a Bohlin C-VOR 120 rotational rheometer equipped with a thermostatic unit (KTB 30). Oscillatory shear conditions were applied during all the rheological tests performed at 37 °C using a shagreened parallel stainless-steel plate apparatus, diameter = 40.0 mm, as measuring device and fixing the gap to 2.3 mm. The mechanical spectra (frequency sweep test, stress = 5 Pa, frequency range: 0.01–10 Hz) and the extension of the linear viscoelastic regime (stress sweep test, frequency = 1 Hz, stress range: 0.1–5000 Pa) were acquired at 37 °C (n = 5). All the hydrogel formulations were characterized and analysed according to previously reported procedures [52].

#### 2.5.3 Dynamic mechanical analysis (DMA)

DMA was performed on the hydrogel groups: GG, GG_HA_Coll_high, GG_HA_MCollB_high and GG_HA_MCollB_high_aligned (Young modulus) (DMA Q800 dynamic mechanical analyser; TA Instruments). Samples were cast as described in the hydrogel preparation section 2.3 (GG, GG_HA_Coll_high, GG_HA_MCollB_high: 35 mm diameter, 3 mm thickness; GG_HA_MCollB_high_aligned 20 mm diameter, 3 mm thickness the SMF was applied during the gelation with the setup described above) and punched after an overnight immersion in PBS 1X at 37 °C to obtain discs with a diameter of 8 mm and a height of 2–3 mm. All the analyses were carried out at 37 °C in PBS 1X submersion. The Young modulus was evaluated in compressive mode and a stress-strain test was performed to obtain the slope of the linear fit in the range from 0 % to 10 % (n = 5). The stress relaxation behaviour was investigated in stress relaxation mode, an initial 10 % compressive strain was quickly applied and held constant for 10 min while the load was recorded as a function of time to evaluate the relaxation of the stress. The relaxation time (τ_1/2_) necessary for the initial stress to be relaxed to half its value during the analysis was determined.

### 2.6 *In vitro* biological evaluation

Murine fibroblast cell line BALB-3T3 Clone A31 (ATCC® CCL-163™), murine monocyte/macrophage cell line RAW 264.7 (ATCC® TIB-71™) and murine myoblast cell line C2C12 (ATCC® CRL-1772™), purchased from American Type Culture Collection (ATCC), were used. BALB-3T3 were cultured in DMEM high glucose supplemented of 10 % CBS and 1 % of pen/strep (100 U/ml - 100 μg/mL), RAW 264.7 were grown in DMEM high glucose supplemented of 10 % FBS and 1 % pen/strep, C2C12 were cultured in DMEM high glucose supplemented of 20 % FBS and 1 % pen/strep and differentiated in DMEM high glucose added of 2 % horse serum and 1 % pen/strep. All the cell lines were cultured in a controlled environment in terms of temperature (37 °C), humidity and CO_2_ (5 %). BALB-3T3 and C2C12 were detached from culture flasks by trypsinization whereas RAW 264.7 were detached by scraping. Then the cells were centrifuged, and the Trypan Blue Dye Exclusion test was performed to assess the cell number and the cell viability. All the procedures were carried out in sterile conditions using a laminar flow hood. Cells were encapsulated into the hydrogels by mixing them using a 1 mL syringe. Then the mixture was quickly loaded into a 1 mL 30G needle syringe and three drops of gel were poured into each well (96 well-plate) containing 200 μL of complete media. C2C12 mixture was loaded into a 1 mL syringe and 70 μL of gel, poured into each well (96 well-plate), sprayed on top with differentiation media and after 10 min of incubation 100 μL of differentiation media were added. Cell concentrations used: BALB-3T3 were cultured at 5.0 x10^5^ cells/mL; RAW 264.7 at 2.0 x10^6^ cells/mL; C2C12 at 6.0 x10^6^ cells/mL.

All the analyses were performed on GG, GG_HA_Coll_high, GG_HA_MCollB_high hydrogel formulations (n = 5).

#### 2.6.1 Viability and proliferation assay

A qualitative analysis was performed to evaluate BALB-3T3 and C2C12 cell viability. LIVE/DEAD® assay was done on day 1, 3 and day 7 following the manufacturer's instructions. Briefly, cells were washed with PBS 1X and incubated with 1.3 μM of Calcein AM and 4 μM of Ethidium homodimer-1 for 15 min at 37 °C and 5 % CO_2_. Live cells stained in green and dead cells in red were acquired by using an Inverted Ti-E Fluorescent Microscope (Nikon).

Presto blue™ assay was used to quantitatively assess cell viability and proliferation, the resazurin-based reagent was incubated according to the manufacturer's instructions for 2 h at 37 °C and 5 % CO_2_. BALB-3T3 were tested on day 1, day 3 and day 7 whereas RAW 264.7 at day 2 of culture. The reagent is converted by living cells in fluorescent resorufin which was detected by using the Fluoroskan™ Microplate Fluorometer (Thermo Fisher Scientific) setting the excitation wavelength equal to 544 nm, whereas the emission wavelength to 590 nm. A hydrogel without cells was used as *blank* and its background value was then removed from all the viability data. The results were plotted for RAW 264.7, whereas BALB-3T3 data were normalized on day 1 and then plotted (n = 5).

#### 2.6.2 Evaluation of gene expression profile

The gene expression profile of RAW 264.7 after 48 h of culture was evaluated to determine the eventual M1 or M2 polarization. As a positive control for M1 activation RAW 264.7 embedded into GG hydrogel formulations were incubated with 1 μg/mL of LPS, which was replaced every 24 h. Total RNA extraction was performed by Tri reagent, followed by the Directzol RNA MiniPrep kit, following the manufacturer's instructions. Then the RNA was quantified and the purity degree was evaluated using the NanoDrop One Microvolume UV–Vis Spectrophotometer (Thermo Scientific). The HighCapacity cDNA Reverse Transcription Kit was used to obtain a single strand cDNA starting from 500 ng of purified RNA, following the manufacturer's instructions. The gene expression was evaluated using TaqMan Gene Expression Assays for TNF-α (Mm00443258_m1), IL-10 (Mm01288386_m1), IL-1β (Mm00434228_m1), and GAPDH (Mm99999915_g1) used as housekeeping gene, it was performed by QuantStudio 1 Real-Time PCR System (Applied Biosystems). Two samples for conditions were processed and three technical replicates were performed. Relative quantification was performed using the comparative threshold (Ct) method (ΔΔCt), where the relative gene expression level equals 2^−ΔΔCt^ [53].

#### 2.6.3 Cell Morphology analysis

At day 7 hydrogels containing C2C12 cells were fixed for 20 min in 4 % PFA, and cells permeabilized using PBS 1X with 0.1 % (v/v) Triton X-100 for 10 min. The cytoskeleton actin filaments were visualized by 40 min incubation of Actin Red 555 ready probes and then the nuclei were counterstained by DAPI (600 nM) for 15 min. The images were acquired by using an Inverted Ti-E Fluorescent Microscope (Nikon).

### 2.7 *In vivo* biological evaluation

#### 2.7.1 Animal Experimentation

Male Wistar Han (Crl:WI/Han) rats (14 animals, age = 10 weeks) were used for hydrogels subcutaneous implantation (GG, GG_HA_Coll_high, GG_HA_MCollB_high). Briefly, the animals were anesthetized by isoflurane inhalation, the hair in the dorsum was removed and the surgical area was prepared with an aseptic technique. 200 μL of each gel formulation was administered through a 30-G syringe in duplicate on the lateral left and right sides. After 1, 3, 7 and 28 days, rats were sacrificed and the hydrogels and surrounding tissues were processed for histological analyses. Potential systemic toxicity was evaluated in the liver, spleen, kidney and lymph nodes. Animal experimentation was carried out at i3S - Instituto de Investigação e Inovação em Saúde animal facility, in accordance with European Legislation on Animal Experimentation through the Directive 2010/63/UE and approved by the institutional animal ethics committee and the Portuguese official authority regulating laboratory animal sciences (DGAV).

#### 2.7.2 Histological analysis

Tissues and hydrogels were collected, fixated in 10 % neutral buffered formalin for 24 h at RT and processed for paraffin embedding. Sequential 7 μm sections were collected, the paraffin removed and the samples hydrated by sequential washes in xylene, ethanol 100 %, ethanol 80 % and MilliQ water. Sections were then stained.

Haematoxylin and Eosin staining was performed following the manufacturer's instructions, briefly the Mayer Haematoxylin added of acetic acid was incubated for 3 min at RT and washed for 10 min in running water followed by a wash in MilliQ water. Then the Eosin Y was incubated for 3 min and washed in MilliQ water.

For Safranin-O staining, Mayer Haematoxylin was added as described above, then 3 min incubation of Fast Green solution was performed followed by 0.1 % v/v acetic acid solution washes. Lastly, Safranin-O was incubated for 5 min and then washed in MilliQ water.

Prussian Blue staining was done by incubating 20 min the Potassium hexacyanoferrate (II) mixed to 20 % v/v HCl and followed by 3 washes in MilliQ water and then Mayer Haematoxylin stain was done as described above.

After all the staining protocols, the slides were dehydrated using increasing ethanol concentrations up to 100 % and a final wash in xylene before the mount.

Part of the hydrogels collected were fixed in PFA 4 %, cryopreserved in increasing scale of sucrose (10 %, 20 %, 30 %) at 4 °C and embedded in OCT. Then the samples were left overnight at −20 °C before proceeding with the sectioning at the cryostat (Histo-Line). Sequential 30–50 μm sections were collected and DAPI staining was performed. The slices were washed for 1 min in running water, incubated in 0.1 % Triton X-100 PBS 1X for 5 min followed by 5 min of incubation with DAPI solution (600 nM), then washed with running water and mounted as described above. The images were acquired by using an Inverted Ti-E Microscope.

### 2.8 Statistical analysis

All the results were plotted ± standard error and statistical analyses were performed by GraphPad Prism Software (Version 8.0). BALB-3T3 proliferation analysis data was analysed by Two-way analysis of variance (Two-way ANOVA), followed by Tukey's Multiple Comparisons test. RAW 264.7 cell viability data was analysed by One-way analysis of variance (One-way ANOVA), followed by Tukey's Multiple Comparisons test. Gene expression data was analysed by both Two-way ANOVA, followed by Tukey's Multiple Comparisons test and Paired Student T-test. Statistically significant differences are reported in the graphs: *p value ≤ 0.05, **p value ≤ 0.01, ***p value ≤ 0.001 and ****p value ≤ 0.001.

## 3 Results and discussion

### 3.1 Synthesis and characterization of magnetic collagen bundles

Collagen type I (Coll) is the most abundant collagen in the human body and serves as the primary structural component of several tissues [54]. It was selected to enhance the biomimetic nature of the proposed system. Coll fibers, owing to their positive charge (+10.2 ± 0.6 mV) could readily interact with negatively charged MNPs (−53.2 ± 1.3 mV). Additionally, due to the Coll fiber dimensions, MNPs could be easily absorbed on the collagen fiber covering the surface, thereby forming magnetic Collagen fibers through electrostatic attractions [55]. MNPs and a Coll liquid suspension were combined using various mass ratios: 1:1, 1:2, and 1:3, respectively. The amount of Coll was gradually increased while keeping the concentration of MNPs constant. This is essential as we require the minimum MNPs concentration necessary to obtain MCollB able to be aligned in a low-intensity SMF. As expected, the results indicated that increasing the amount of Coll led to a greater quantity of Coll bound to MNPs, with the highest binding observed at 0.375 mg of Coll in 1 mL of MCollB (Fig. 1 A). Considering the non-negligible difference in Collagen content between the 1:1 (MCollB_low) and 1:3 (MCollB_high) ratios, we decided to conduct a more in-depth investigation of both ratios to assess whether a higher concentration of Collagen was eliciting different magnetic effects. In fact, these fibers should be able to be remotely aligned by applying a static low-intensity Magnetic Field (SMF) to create "pathways" that facilitate cell adhesion and alignment within the hydrogel matrix. The magnetic properties of MCollB were assessed using AC magnetometry. This technique has been widely employed to investigate encapsulation strategies [56,57] and to characterize magnetization cycles of magnetic nanoparticles dispersed in liquids [58,59] or biological matrices [60]. Fig. 1 B displays the AC hysteresis loops obtained for free MNPs and for MCollB_low and MCollB_high. Magnetization cycles exhibit some alterations due to dynamic phenomena [61], but the maximum magnetization values decrease when MNPs are dispersed within collagen. The immobilization of MNPs within collagen may contribute to dipolar interaction phenomena, resulting in demagnetization [62] of MCollB structures and no differences were detected among the MCollB_low and MCollB_high groups. This magnetic characterization in liquids confirms that MCollB are promising candidates as magnetic-responsive elements.

Examining the MCollB using FEG-SEM analysis revealed a pronounced interaction between the collagen fibers and the MNPs (Fig. 1C and D). The collagen appears to be nearly completely enveloped by the MNPs. Additionally, EDS analysis demonstrated the presence of collagen beneath the MNPs in areas where the collagen was not visibly apparent (Fig. 1E and F). The ability of MCollB to obtain an aligned conformation was then evaluated through microscope analyses. MCollB_low and MCollB_high were resuspended in a liquid, and a drop from each group was placed between two magnets and subjected to a 55 mT SMF. The low-intensity external magnetic field was generated with two Neodymium magnets placed 4 cm apart. The north and south poles of each magnet were facing each other, and the magnetic field in the central region (2 cm from each magnet) was simulated in COMSOL Multiphysics Software (version 5.4) (Fig. 1 G) and confirmed with a magnetometer.

In both samples, MCollB alignment was achieved very rapidly (<2 s), as confirmed by optical microscopy, with no noticeable macroscopic differences between the groups, given that the MNPs content was identical (Fig. 1 H and K). SEM analysis further confirmed a well-oriented distribution of MCollB achieved through the application of SMF, in contrast to the random distribution observed without SMF. Additionally, SEM images depicted an aggregation of collagen bundles, partly attributed to the drying process required for analysis (Fig. 1 J and L).

The presence of collagen bundles was also confirmed by AFM imaging; indeed, from the analysis, of AFM topography images, collagen bundles with typical dimensions 2–5 μm could be appreciated (Fig. 1M–T). In the absence of SMF, collagen fibers were clearly arranged in a mat fashion, with the fibers settling on the substrate's surface, thus suggesting an inhomogeneous interaction between the MNPs and the collagen (Fig. 1 Q and R). Nonetheless, the interaction resulted strong enough to rearrange both MNPs and collagen fibers to build up MCollB through the application of a low-intensity SMF, as demonstrated by the absence of bare Coll fibers around the aligned MCollB (Fig. 1 S and T). In the MCollB_high group, where the relative concentration of Collagen is higher, few collagen fibers emerging from the bundle were still visible (Fig. 1 T, indicated by red arrows).

In the MCollB_high group, where the relative concentration of Collagen is higher, few collagen fibers emerging from the bundle were still visible (Fig. 1 T, indicated by red arrows). In the literature, several attempts have been made to achieve remote magnetic alignment of various nanostructures in order to impose a well-defined architecture on biomaterials. For instance, collagen fibers, cellulose nanocrystals, carbon nanotubes, or collagen-silica bionanocomposites have been aligned using a Static Magnetic Field (SMF). However, these methods often require a strong magnetic field in the range of tesla (T), which is not easy to manage [[63], [64], [65], [66]]. To address these limitations, Magnetic Nanoparticles (MNPs), already studied for numerous bio-applications such as anti-cancer drug delivery, hyperthermia treatment for solid tumors, imaging and cell labeling, and production of scaffolds for the regenerative purpose have been investigated due to their chemical stability, ease of functionalization, and high saturation magnetization, especially in the case of iron oxide-based MNPs [40]. These properties allow for a significant reduction in the required magnetic flux density [67]. Nevertheless, the biocompatibility and clearance of these MNPs remain highly debated in the context of regenerative medicine approaches [68,69]. In this study, we confined MNPs to collagen fibers to enhance the biomimicry of our system and reduce any potential adverse effects of MNPs. Furthermore, our initial analyses demonstrated the ability of MCollB to achieve well-defined alignment in the presence of a few millitesla (mT) magnetic fields. The straightforward formulation of MCollB, in contrast to the more complex and expensive preparation of magnetic electrospun or 3D printed fibers, makes it easy to handle and cost-effective [35,37,43,44].

The combination of all the aforementioned features of MCollB confirms their potential as the magnetic-responsive components of our injectable hydrogel system.

### 3.2 Hydrogel formulation and characterization

**Fig. 2:**
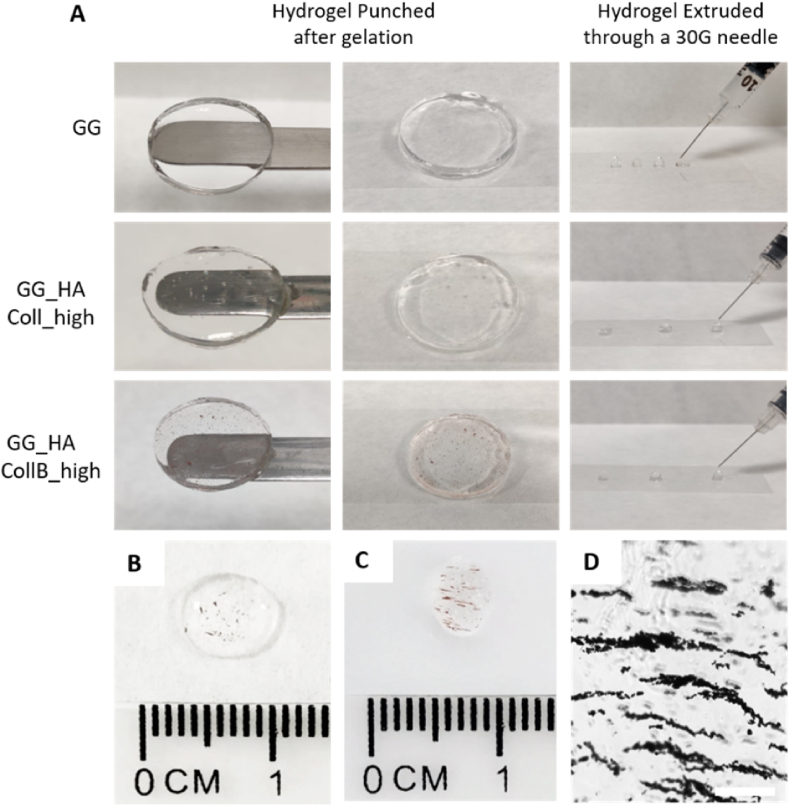
Hydrogel disks and drops of the different groups extruded through a 30G needle (A). Proof of concept of the aligned architecture: after the injection of MCollB_high group (B), SMF is applied and the alignment was clearly visible (C). Aligned structure is maintained after the gelation (D). Scale bar 200 μm.

Gellan gum is a bacterial-derived polysaccharide, renowned for its biocompatibility and biodegradability. Widely employed as a food additive, it is valued for its remarkable gelling capability [70,71]. Specifically, GG's gelling behavior depends on temperature and the presence of cations in the solution (e.g., PBS, cell culture media, biological fluids), leading to the creation of stable and thermoreversible gels [72]. To create an injectable system stable at 37 °C, 0.1 % w/v sodium citrate was added to the GG solution during solubilization. The resulting hydrogel could be easily injected through a 30G needle, an appropriate size for minimally invasive surgery, and gelation occurred almost instantly upon contact with PBS 1X (Fig. 2).

While GG-based hydrogel can be readily obtained, it is still relatively underutilized in the biomedical field, with limited studies exploring its potential for biomolecule/cell encapsulation, delivery, or regenerative medicine applications [[73], [74], [75]]. This is primarily due to the fact that GG, like alginate, is a relatively inert biomaterial [76]. To better mimic the human extracellular matrix (ECM) [77,78] and enhance the bioactivity of the hydrogel, we used GG as the backbone material supplemented with hyaluronic acid (HA, 0.3 % w/v) and Coll (bound to MNPs, forming MCollB as previously described). Both HA and Coll are important components of the ECM and play key roles in regulating cell differentiation, migration, angiogenesis, and the inflammatory response [79,80]. Additionally, Coll in this context serves the additional purpose of creating a defined architecture during the gelation process when subjected to a SMF. The results demonstrated that the obtained hydrogel (GG_HA_MCollB) was stable and, like the GG hydrogel, and could be easily extruded to form a transparent hydrogel that could also be punched after gelation (Fig. 2 A). The presence of MCollB, as opposed to only "nude" Collagen (used as a control group), did not impede extrusion through a 30G needle (Fig. 2 A). This feature makes it particularly advantageous compared to other promising injectable systems where the injection through fine needles may be compromised due to material viscosity [[81], [82], [83]]. In addition, unlike some other hydrogels where gelling agents require specific conditions like exposure to UV light for the gelation process, GG provides a more versatile option that takes advantage of physiological cations offering a flexible and practical alternative to other cross-linking methods, especially when the limitations of light penetration or cross-linker toxicity need to be considered [84,85]. Furthermore, the hydrogels exhibited less than 35 % degradation after 80 days in PBS 1X at 37 °C, indicating good stability of the formulation (Fig. SI 2).

The feasibility of creating a contactless magnetically responsive hydrogel that allows for remote generation of an anisotropic architecture was demonstrated as a proof of concept by extruding the GG_HA_MCollB hydrogel through a 30G needle while applying a low-intensity SMF. A rapid (∼10 min) and clear alignment of MCollB within the matrix, with no differences between low and high Coll content, was macroscopically observed (Fig. 2 B and C; Video SI 1 and 2). The achieved aligned structure was maintained after gelation, as shown in Fig. 2 D.

Supplementary data related to this article can be found online at https://doi.org/10.1016/j.mtbio.2024.101110

The following are the Supplementary data related to this article.

Video S1 Video S1

Video S2 Video S2

Video S3 Video S3

**Fig. 3:**
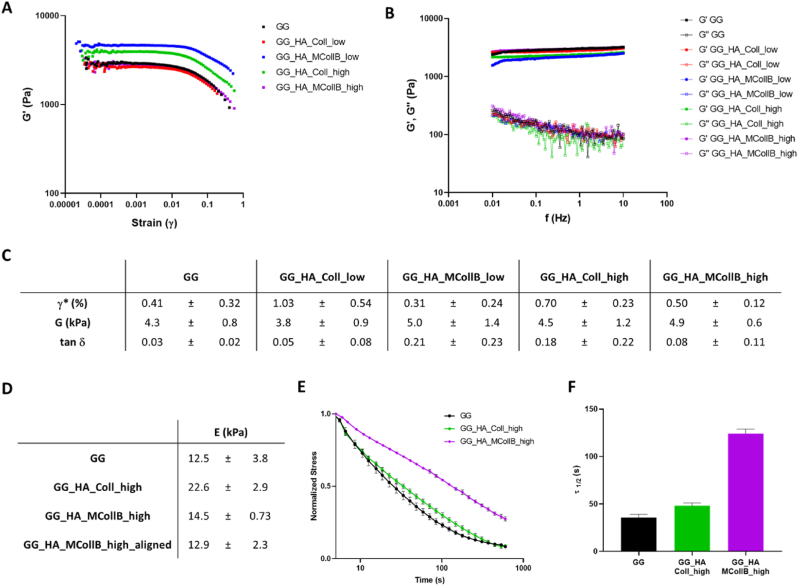
Mechanic properties evaluation. (A–C) Rheological characterization, (A) Stress sweep test (f = 1Hz). (B) Frequency sweep test (stress = 5 Pa), (C) Viscoelastic properties evaluation. Critical strain (γ*), shear modulus (G) and tan δ (G’’/G′). (D–F) Dynamic mechanical analysis hydrogels characterization. (D) Young's Moduli. (E) Stress relaxation curves were obtained by applying an initial 10 % strain from which was calculated the relaxation (τ_1/2_) value (F).

Considering the ultimate regenerative purpose of the proposed hydrogel, the replication of ECM mechanical properties is clearly a crucial design parameter for regulating cell behavior. The mechanical, structural, and chemical composition of the surrounding ECM are indeed key regulators of intracellular processes and cell behaviors, including adhesion, spreading, proliferation, and differentiation [86]. In this regard, the viscoelastic properties of the hydrogels were assessed under oscillatory shear conditions. The linear viscoelastic regime (LVR) was determined through a stress sweep test (Fig. 3 A), which revealed the critical strain, i.e., the point at which strain softening occurs, falling within the range of 0.27–1% (Fig. 3 C; Table SI 1). This suggests that our system exhibits good resistance to applied strain. In frequency sweep tests within the LVR, the storage modulus (G′) consistently exceeded the loss modulus (G″) for all formulations, indicating that the resulting systems behave as classic viscoelastic gels (Fig. 3 B; Fig. SI 2). The shear modulus (G), which corresponds to the stiffness of hydrogels under constant stress at small deformations, was calculated using the Maxwell model and ranged from 3 to 9 kPa, indicating a robust mechanical response for all formulations (Fig. 3 C; Table SI 1). Furthermore, the tan δ (G''/G′) values obtained (Fig. 3 C; Table SI 1) were all <1, confirming the predominance of the elastic component over the viscous one [87]. Taken together, these findings suggest that no significant alterations in the physical properties of the gels or gelation kinetics occurred following the addition of HA, Collagen, and MCollB to our GG matrix.

Given that no differences related to Coll content were observed in the above results, and considering the biomimetic nature of our system, we chose to proceed with further investigations using only the hydrogels containing the highest collagen quantity (GG_HA_MCollB_high) and its corresponding control (GG_HA_Coll_high), along with the GG matrix alone as a control.

To assess whether the addition of MCollB to the hydrogel altered the overall matrix stiffness, we conducted a dynamic mechanical analysis. A stress-strain test in compressive mode was performed to determine the Young's Modulus (E), which is represented by the slope of the linear fit in the range from 0 to 10 % strain (Fig. 3 D). To create a more physiologically relevant environment, all tests were conducted at 37 °C in PBS 1X. The obtained Young's Moduli fell within the range of 12.5–22.6 kPa, which is comparable to human soft tissues such as neural tissues, thyroid, spleen, and muscle [88,89]. Moreover, there is no difference between GG_HA_MCollB_high aligned and not meaning that the alignment does not impact the mechanical properties of the final system. Stiffness is an important material design parameter that should also be correlated with stress relaxation, as human living tissues are viscoelastic. Stress relaxation involves the material releasing stored energy over time after the application of a defined strain. This phenomenon allows the material to deform, enabling cell polarization, migration, or spreading [90]. As illustrated in Fig. 3 E and F, our hydrogels exhibit an increased stress relaxation behavior when HA and Collagen are added to GG. Notably, the presence of MCollB_high statistically prolongs the relaxation time (τ_1/2_), which represents the time required for the initial stress to relax to half of its value (Fig. 3 F) [4,91]. The GG_HA_MCollB_high hydrogel displayed a stress-relaxation curve closely resembling native muscle behavior (Fig. 3 E) [86], falling within the range previously reported for alginate-based hydrogels that have the capacity to support cell spreading [90].

The developed formulations are easy to handle and user-friendly due to their capacity to transform into a stable, viscous liquid that readily gels in the presence of cations, eliminating the need for precursor mixing, as is often required in the literature [92,93].

Furthermore, it can be affirmed that the formulated hydrogels exhibited favorable mechanical strength and properties similar to soft biological tissues. The inclusion of MCollB_high enhanced their stress relaxation behavior, rendering them suitable for minimally invasive regenerative medicine applications via injection.

### 3.3 *In vitro* biological evaluation

**Fig. 4:**
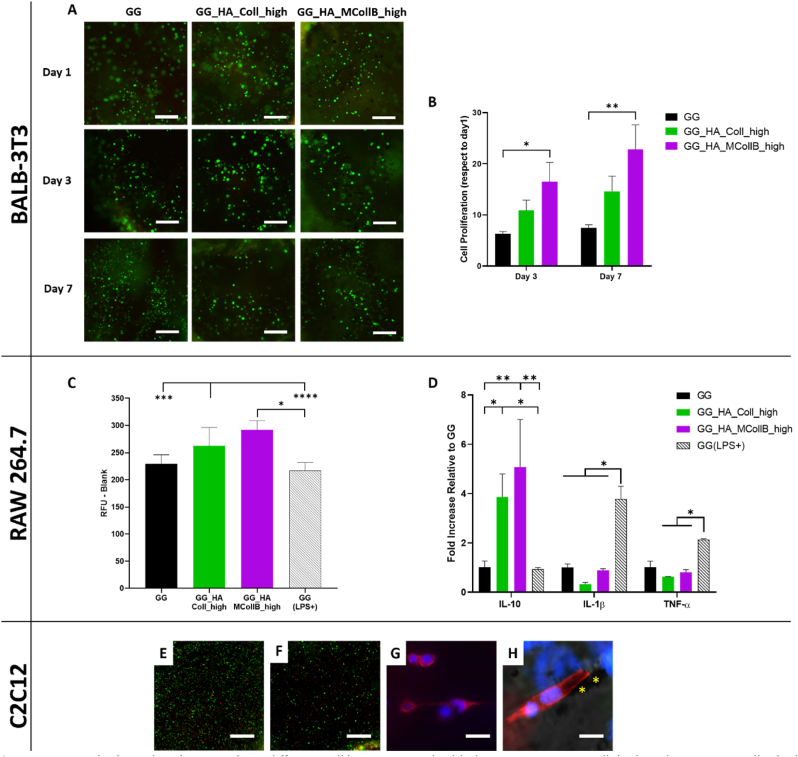
In vitro biological evaluation. Three different cell lines were embedded into GG, GG_HA_Coll_high and GG_HA_MCollB_high hydrogels: BALB-3T3 cells (A) and (B); RAW 264.7 cells (C) and (D), C2C12 (E–H). (A) BALB-3T3 LIVE/DEAD® assay to assess cell viability at 1, 3 and 7 days. (B) BALB-3T3 proliferation evaluation up to 7 days of culture. (C) RAW 264.7 cell viability analysis at 48 h. (D) RAW 264.7 gene expression profile relative to M1 (IL-1β and TNF-α) and M2 (IL-10) phenotype at 48 h. (E–F) C2C12 LIVE/DEAD® assay at day 1 and day 7 (F). (G–H) C2C12 cell morphological analysis staining, (H) merge of brightfield, actin and dapi; * indicate MCollB. Scale bars: (A, E, F) 250 μm; (G) 20 μm; (H) 15 μm. Statistical analysis: *p value ≤ 0.05, **p value ≤ 0.01, ***p value ≤ 0.001 and ****p value ≤ 0.001.

An *in vitro* evaluation was conducted to assess the biological behavior of the hydrogels. Specifically, a murine fibroblast cell line (BALB-3T3) was selected for this study, as fibroblasts are typically present in the ECM, and BALB-3T3 cells are commonly used as a standard cell culture model for initial biomaterial screening. The cells were encapsulated within the hydrogels, and their viability and proliferation were assessed at three-time points (day 1, day 3, and day 7). The qualitative LIVE/DEAD® assay revealed a notably high ratio of viable cells, with no significant differences observed among the groups, indicating the absence of cytotoxicity in all tested hydrogels (Fig. 4 A).

The results of cell proliferation highlight the capacity of our systems to support cellular growth over time, with GG_HA_MCollB_high exhibiting a higher proliferation rate compared to GG_HA_Coll_high and GG groups. These findings confirm that the addition of HA and Collagen enhances the hydrogel's bioactivity, stimulating cell proliferation (Fig. 4 B). Furthermore, given the significantly higher proliferation observed in GG_HA_MCollB_high, it suggests that the presence of MNPs may play a pivotal role. It is well established that MNPs can influence cell behavior upon internalization [94,95], and notably, they can enhance cell proliferation [96]. Since MNPs are bound through weak interactions, we can speculate that they may be readily accessible to cells and taken up, thereby stimulating cell metabolism (further studies will be necessary to confirm this hypothesis).

Furthermore, to elucidate the immunological profile of the hydrogels, an analysis of murine macrophages (RAW 264.7) was also conducted [97,98]. The pivotal role of macrophages in orchestrating adverse immune responses against foreign materials, often referred to as the foreign body response, has been well-established over many decades [97,99]. When implanted, biomaterials are frequently recognized as foreign bodies by the immune system, triggering detrimental immune responses. Macrophages exhibit dynamic and plastic phenotypes that adapt to changes in the microenvironment, including the polarization from the pro-inflammatory M1 phenotype to the pro-regenerative M2 phenotype [100]. The assessment of macrophage viability helps identify potential immune cell cytotoxicity in response to a material. RAW 264.7 cells were encapsulated within the hydrogels, and after 48 h, an increase in cell numbers was observed in the hydrogels GG_HA_MCollB_high and GG_HA_Coll_high compared to the GG group and the LPS-induced M1 macrophage activation group, GG (LPS+), which served as a positive control (Fig. 4 C). This increase in macrophage proliferation can be attributed to the M2 activation phase, well-known for promoting cell proliferation and tissue repair compared to the M1 activity, which inhibits cell proliferation and causes tissue damage [101]. In fact, gene expression analysis of the typical M1/M2 genes (IL-1β, TNF-α: M1; IL-10: M2) revealed a statistically significant over-expression of IL-10 in GG_HA_Coll_high and GG_HA_MCollB_high compared to GG and GG (LPS+) (Fig. 4 D). These findings are consistent with existing literature, as it is known that HA triggers M2 activation [102], while GG alone is considered an inert material. Additionally, the absence of any difference between GG_HA_MCollB_high and GG_HA_Coll_high indicates that the addition of MNPs to the hydrogel did not elicit an inflammatory response. A preliminary study was conducted to further validate the potential of the proposed aligned hydrogel. Muscle cells were embedded in the GG_HA_MCollB_high hydrogel and exposed to a 55 mT SMF for the gelation period (10 min). Cell viability confirmed a high number of live cells (Fig. 1 E–F), and notably, the initial alignment of the cells along the hydrogel's directional properties was observed (Fig. 1 G–H, VIDEO SI 3). The *in vitro* results emphasize that the novel magnetic injectable hydrogel provides a favorable microenvironment capable of sustaining the viability and proliferation of fibroblasts (selected as the cell model) while also polarizing macrophages toward a pro-regenerative phenotype, thereby facilitating the tissue regeneration process.

### 3.4 *In vivo* biological evaluation

**Fig. 5:**
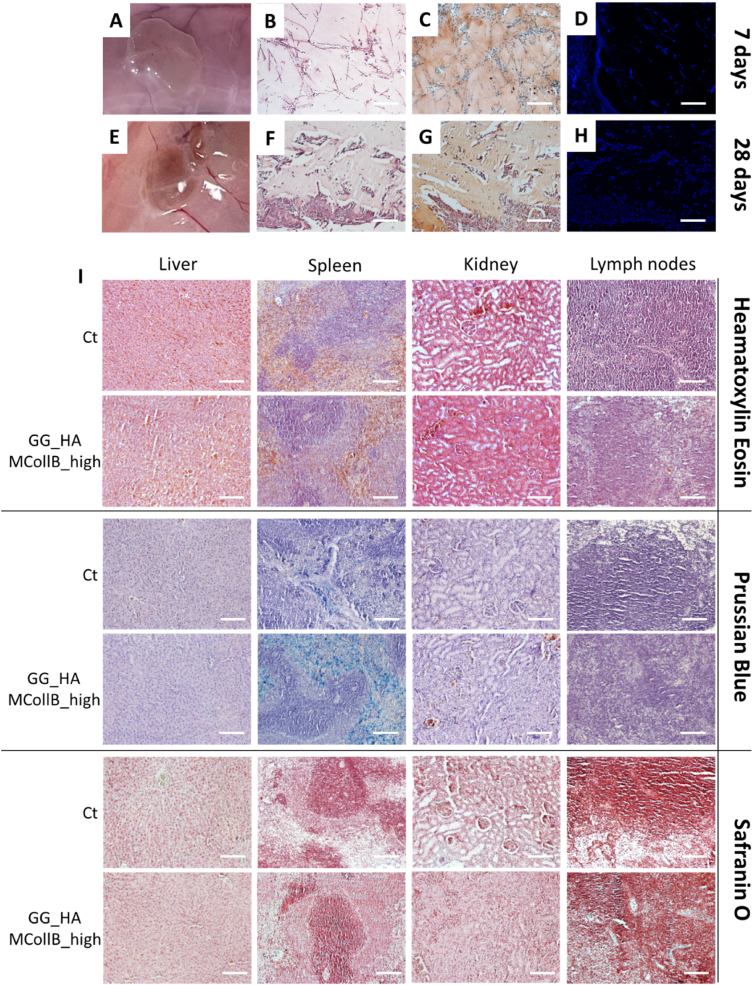
*In vivo* biological evaluation. Hydrogel explant after 7 days and 28 days (A, E). Haematoxylin and eosin (B, F) safranin O (C, G) staining and cell nuclei stained with DAPI (D, H) on hydrogel sections of GG_HA_MCollB_high formulation. Haematoxylin and Eosin, Prussian blue and Safranin O staining were performed on organs explanted at day 28 (I). Scale bars 200 μm.

To assess how well *in vitro* observations could be translated into an *in vivo* context, a pilot study was conducted by subcutaneously injecting our magnetic hydrogels into rats using a 30G needle. Gelation occurred very rapidly (∼2 min) due to the animal's body temperature and the presence of cations in the subcutaneous tissue. Macroscopic evaluations conducted at 1, 3, 7 and 28 days post-injection revealed that all the hydrogels remained in their intended positions, with no evidence of edema, infection, or tissue necrosis (Fig. 5 A, E; SI 3 A). The volume of the hydrogel explant at day 28 is relatively reduced compared to day 7 (Fig. 5 A, E; SI 3 A), indicating that a substantial portion of the hydrogel components remains localized at the injection site, in line with the stability data obtained in PBS at 37 °C up to 80 days. Numerous cells were detected within the hydrogels, indicating their permeability to resident cells that colonize the scaffolds, an essential factor for tissue regeneration (Fig. 5 B, C, D, F, G, H and Fig. SI 3B). Furthermore, histological analysis did not reveal any pathological changes in the harvested organs after 7 and 28 days attributable to the presence of the hydrogels in the animals (Fig. 5 I; SI 3C). There were also no significant accumulations of iron. The lymph nodes, liver, and kidneys exhibited no deviations from the control group, with only minor iron accumulation observed in the spleen, suggesting partial degradation of the MNPs. This observation is consistent with numerous studies indicating that the accumulation of MNPs in the spleen is higher compared to other tested organs, yet without significant adverse effects [[103], [104], [105]].

While these are preliminary results, they are quite encouraging, suggesting that the magnetic hydrogel may be suitable for further comprehensive *in vivo* studies, including the application of a static magnetic field *in situ.*

## 4 Conclusions

In summary, we developed an injectable bioactive hydrogel fully composed of natural polymers without any chemical crosslinkers. Capable of being aligned using a low-intensity static magnetic field. This comprehensive system exhibited mechanical properties similar to human soft tissues, demonstrated high biocompatibility, and supported cellular growth. Additionally, it promoted the activation of the pro-regenerative (M2) macrophage phenotype. A preliminary study using muscle cells has shown the ability of the cells to align along the anisotropy of the material. The *in vivo* evaluation confirmed the injectability of the system through a thin 30G needle, its ability to rapidly gel *in situ* (within less than 2 min), and its biocompatibility, as evidenced by the absence of toxicity. The proposed injectable hydrogel aims to be a versatile tool for regenerative medicine in the context of aligned tissues. Depending on the specific disease to be treated, it can be further enhanced with modular components such as small molecules (e.g., drugs, growth factors), extracellular vesicles, and tissue-specific ECM elements. In conclusion, it holds immense promise for advancing regenerative medicine and therapeutic interventions tailored to harness the inherent potential of tissues in a controlled and targeted manner.

## CRediT authorship contribution statement

**Arianna Rossi:** Writing – review & editing, Writing – original draft, Methodology, Investigation, Formal analysis. **Franco Furlani:** Writing – review & editing, Methodology. **Giada Bassi:** Writing – review & editing. **Carla Cunha:** Writing – review & editing, Methodology, Funding acquisition. **Alice Lunghi:** Writing – review & editing, Methodology. **Filippo Molinari:** Writing – review & editing. **Francisco J. Teran:** Writing – review & editing, Supervision, Funding acquisition. **Florigio Lista:** Writing – review & editing. **Michele Bianchi:** Writing – review & editing, Supervision. **Anna Piperno:** Writing – review & editing, Supervision, Funding acquisition. **Monica Montesi:** Writing – review & editing. **Silvia Panseri:** Writing – review & editing, Writing – original draft, Supervision, Project administration, Funding acquisition, Formal analysis, Conceptualization.

## Declaration of competing interest

The authors declare that they have no known competing financial interests or personal relationships that could have appeared to influence the work reported in this paper.

## Data Availability

Data will be made available on request.
