# Supplementary material for: Contactless magnetically responsive injectable hydrogel for aligned tissue regeneration
Source: Mater Today Bio. 2024 Jun 3;27:101110. doi: 10.1016/j.mtbio.2024.101110 (PMC11360152; doi:10.1016/j.mtbio.2024.101110)
Supplement: Multimedia component 1 [file mmc1.docx]

**Supplementary Information.**

**
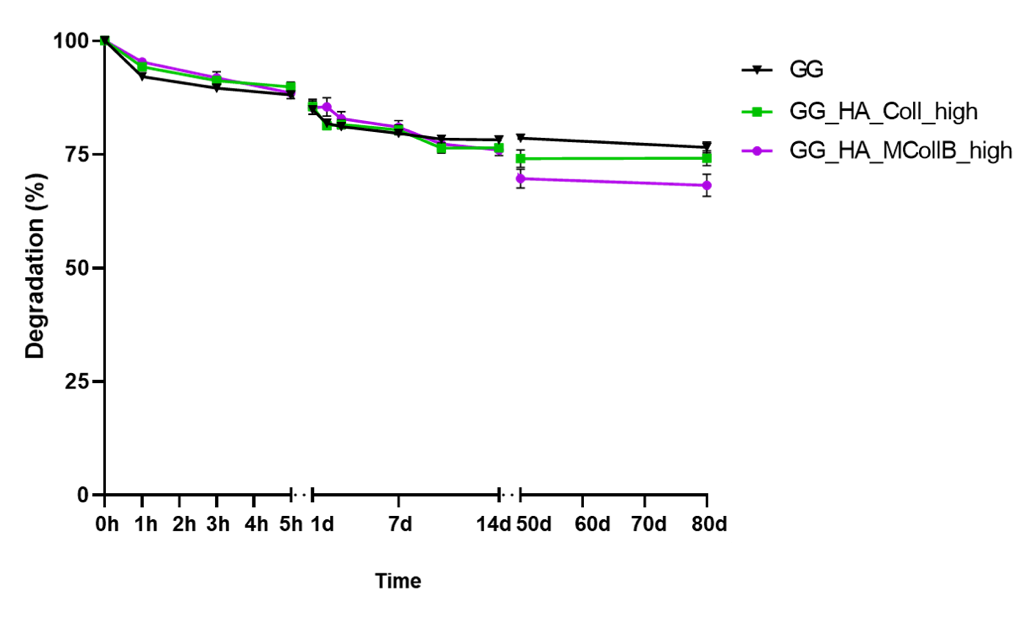
**

**Fig. SI 1.** Stability evaluation. Hydrogels were weighted at different time points, data were plotted respect to day 0 and are reported as mean ± SD.


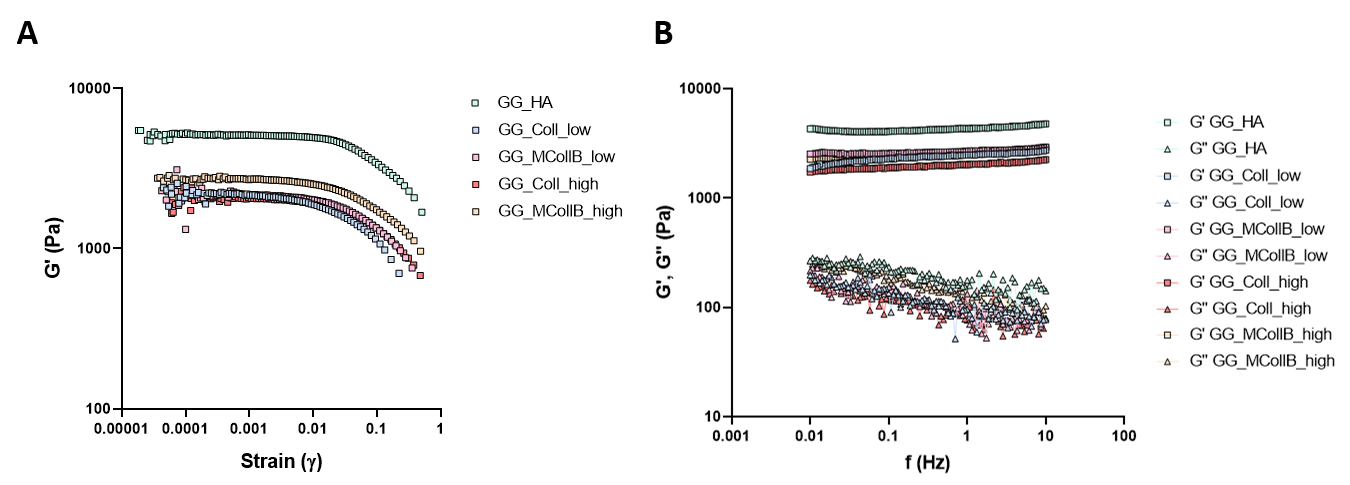


**Fig. SI 2.** Rheological characterization. (A) Stress sweep test (f = 1Hz). (B) Frequency sweep test (stress = 5 Pa).


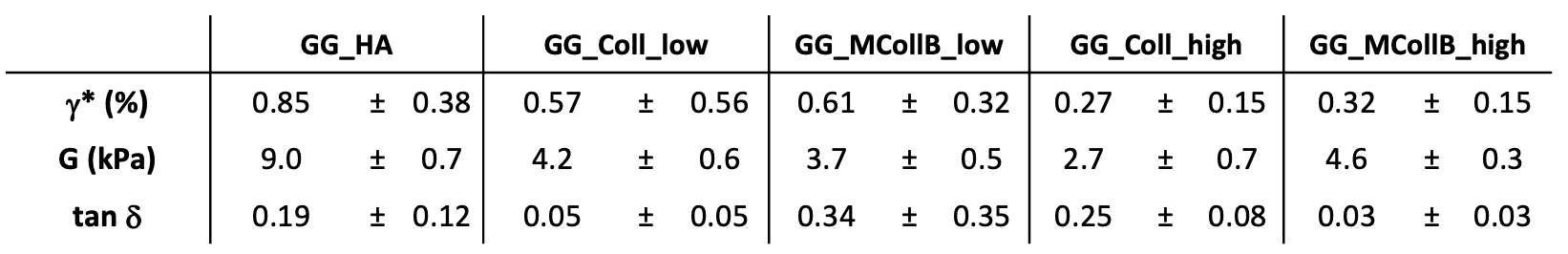


**Table SI 1**. Viscoelastic properties evaluation. Critical strain (γ*), shear modulus (G) and tan δ (G’’/G’).


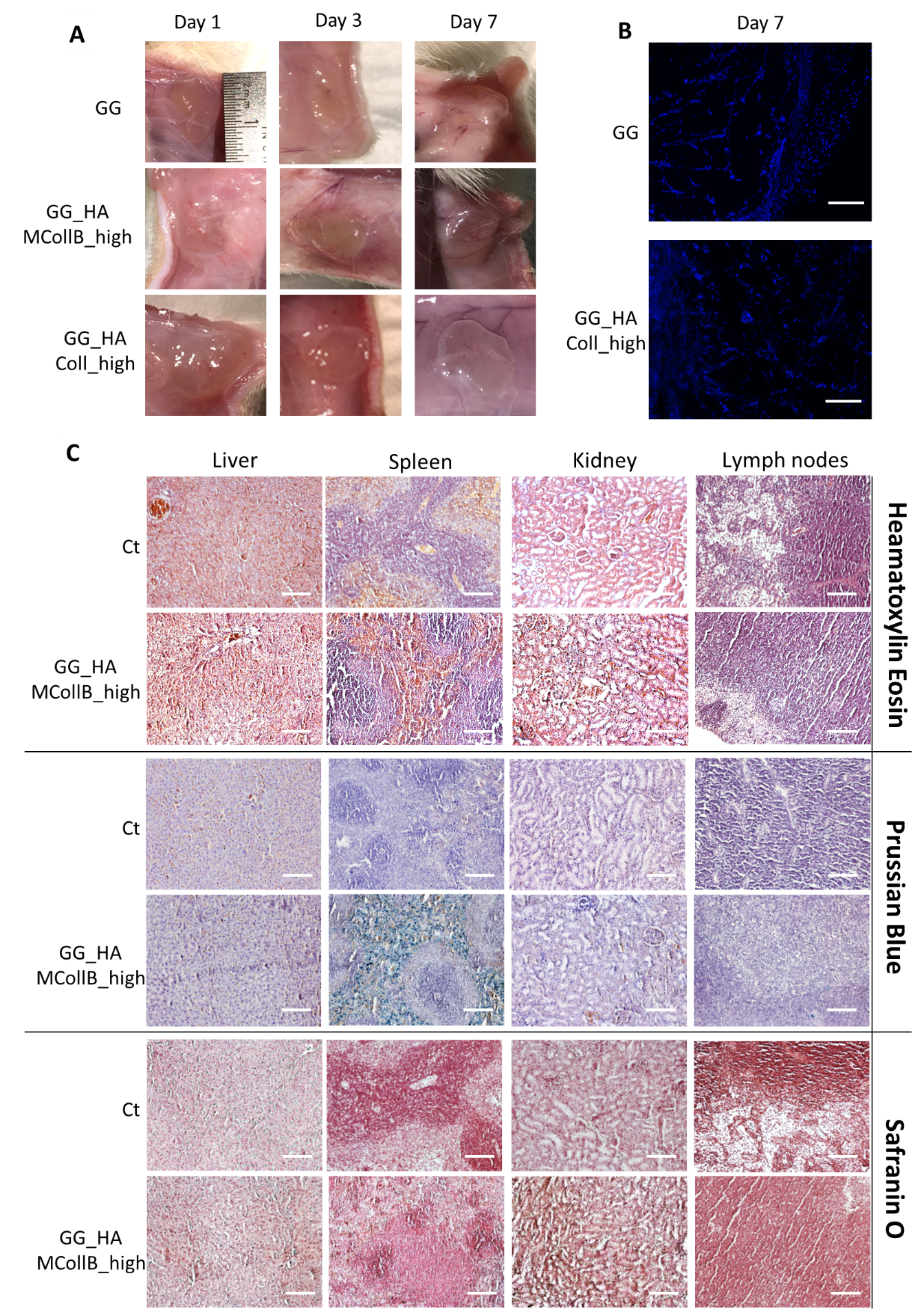


**Fig. SI 3.** In vivo biological evaluation. (A) Hydrogel explant after 1, 3 and 7 days. (B) Cell nuclei staining with DAPI on hydrogel sections at day 7. (C) Haematoxylin and eosin, Prussian blue and Safranin O staining were performed on organs explanted at day 7. Scale bars 200 μm.
